# Facilitating improvements in young people’s social relationships to prevent or treat depression: A review of empirically supported interventions

**DOI:** 10.1038/s41398-021-01406-7

**Published:** 2021-05-21

**Authors:** Kate Filia, Oliver Eastwood, Sarah Herniman, Paul Badcock

**Affiliations:** 1 Melbourne School of Psychological Sciences, The University of Melbourne, Melbourne, Victoria 3010 Australia; 2 Orygen, Melbourne, Victoria 3052 Australia; 3 Centre for Youth Mental Health, The University of Melbourne, Melbourne, Victoria 3052 Australia

**Keywords:** Health sciences, Scientific community

## Abstract

Interpersonal difficulties are often implicated in the onset of depressive disorders, and typically exacerbate depressive symptoms. This is particularly true for young people, given rapid changes in, and the increased importance of, their social relationships. The purpose of this narrative review was to identify empirically supported interventions that aim to prevent or treat depression in young people by facilitating improvements in their social environment. We conducted a search of controlled trials, systematic reviews and meta-analyses of such interventions, published between 1980 and June 2020. Our literature search and interpretation of results was informed by consultations with clinical experts and youth consumers and advocates. A number of promising approaches were identified with respect to prevention and treatment. Preliminary evidence was identified suggesting that school- and Internet-based approaches present a viable means to prevent the worsening of depressive symptoms in young people. Notably, delivering interpersonal psychotherapy—adolescent skills training (IPT-AST) in schools appears to be a promising early intervention strategy for young people at risk of full-threshold depressive disorder. In terms of treating depressive disorders in young people, there is strong evidence for the efficacy of interpersonal psychotherapy for adolescents (IPT-A), and preliminary evidence in favour of attachment-based family therapy (ABFT). Results are discussed with respect to recommendations for future research and practice.

Depression is intimately tied to problems in the social world. From an evolutionary perspective, it has been proposed that depression is a response to interpersonal stressors, born from our need to successfully participate in groups^1–3^. Consistent with this, the first onset of disorder is often brought about by deleterious interpersonal experiences and many symptoms and behaviours observed in depression speak directly to the importance of social contexts^4,5^. For example, loneliness is a known risk factor for depressive symptoms^6^, and individuals who are depressed tend to be highly sensitive to threatening social information, display increased reassurance-seeking and social withdrawal, and often report low self-confidence and self-worth^1,7^. This can result in a vicious cycle, with low self-worth leading to further social avoidance, in turn reinforcing negative self-perceptions and perpetuating depressive illness^8–10^. Improving and strengthening ties with the social environment can aid in breaking this cycle, contributing to improvements in depression. Indeed, social support and a sense of belonging are widely recognised protective factors against depression^11–13^. Altogether, it seems clear that facilitating improvements in social relationships is critical for intervention efforts.

This is particularly true for adolescents and emerging adults. Adolescence is a critical period where social milestones are key developmental priorities^14^. During this period, significant importance is placed on a wide range of social relationships, including those with parents, siblings, peers and romantic partners, along with authority figures such as teachers and employers. Improving social skills and subsequent social interactions can lead to more robust interpersonal relationships and increased support from individuals and groups. At the same time, young people can experience painful losses and challenging social circumstances that increase risk for depression, such as first romantic break-ups, problems with peers, familial conflict as growing independence is exerted and challenges associated with transitioning from school to employment^15,16^. At this critical stage of social identity formation, fostering supportive relationships provides lifelong resources to not only prevent or manage depressive illness, but aid in mitigating the long-term health and economic consequences of experiencing depressive symptoms.

Given that problems in social relationships can be targeted and modified, prevention or treatment approaches explicitly targeting such issues present a promising opportunity for health professionals and policy makers to make a meaningful difference to young people at risk of, or experiencing, depression. This is particularly significant when considering that the onset of depression commonly occurs during adolescence, with age of onset peaking during the early-mid 20s^17,18^. Intervening during this time is therefore critical for the prevention and management of early illness. With this in mind, the aim of this narrative review was to identify best-practice, evidence-based approaches that aim to prevent or treat depression in young people by facilitating improvements in social relationships.

## Methods

### Stakeholder engagement

To ensure the outcomes of our review were meaningful and translatable, we partnered with clinical psychologists (*n* = 3), psychiatrists (*n* = 2) and youth consumers and advocates (*n* = 17). To aid in refining our search strategy, and to ensure we focused on areas relevant to young people, and clinicians working with young people, we conducted one-on-one interviews with clinicians, and stakeholder consultation sessions with young people from the Youth Research Council at Orygen (a specialist, early intervention mental health service for young people aged 15–25 years located in Melbourne, Australia).

These consultations helped us identify promising interventions, and with the perspectives of young people in mind, guided our interpretation of the literature. Following synthesis of the literature, we conducted additional sessions during which we presented our findings and obtained again the unique perspectives and preferences from our expert stakeholders. These perspectives contributed to our reporting of results and discussion.

### Literature search

Our literature search was conducted using the ‘Evidence Finder tool’, a search engine jointly developed by Orygen and headspace (National Youth Mental Health Foundation), which enables users to identify peer-reviewed meta-analyses, systematic reviews and controlled trials on prevention and treatment approaches for young people experiencing mental ill-health^19^. This tool was developed as part of an evidence mapping project and is updated annually; the methodology used in conducting these evidence maps is detailed in Hetrick et al.^20^, and detailed search terms for the Evidence Finder tool are available in De Silva et al.^19^. The Evidence Finder tool has been used in a number of reviews and evidence mapping projects to date (e.g., refs. ^19,21–24^).

At the time of our search, the Evidence Finder contained literature published between 1980 and June 30, 2020, extracted from Embase, MEDLINE, PsycInfo and the Cochrane Library. The Evidence Finder was searched using terms detailed in Supplement 1. Additional articles were identified through reference list screening and targeted searches. For inclusion, studies (i.e., controlled trials, systematic reviews and/or meta-analyses) had to report on interventions designed to prevent or treat depression in young people (with a mean, median or age range of 14–24 years inclusive) and have an explicit social orientation or at least one dedicated component or module that aimed to improve a young person’s social skills or relationships. To maximise inclusiveness, we did not limit our search to any particular form(s) of social relationship. Participants in treatment studies must have had a DSM (III-TR or later) or ICD (ninth edition or later) defined depressive disorder. Studies were required to include a validated measure of depression and be published in English.

## Results

### Stakeholder consultation, search results, and study selection

During interviews with clinical experts, the most frequently endorsed treatment to prevent or treat youth depression was interpersonal psychotherapy (IPT). During interviews with youth consumers and advocates, informal ways to foster social connectedness and perceived social support were emphasised as most important, and unequivocally preferred to more formal strategies that improve social skills. Thus, in addition to the search terms developed with the medical librarian, terms pertaining to IPT, social connectedness and perceived social support were included in the search strategy.

After screening all resulting titles and abstracts for broad relevancy (*k* = 1766), full texts were screened against eligibility criteria (*k* = 512), with 140 meeting such criteria. Bearing in mind discussions from expert consultations, the investigators discussed the remaining articles (*k* = 140) and arrived at a consensus concerning those to include for the purposes of the current report (*k* = 44). An additional nine studies were identified via reference list screening and targeted searching. Of this final pool of included studies (*k* = 53), 40 were controlled trials, 2 were systematic reviews and 11 were systematic reviews with meta-analysis. At the authors’ discretion, eight additional articles were also included to provide supplementary information on the identified interventions.

Synthesis of the included studies revealed a number of prevention approaches, including school-based and online approaches, and treatment approaches, including interpersonal and family-focussed psychotherapy. Despite youth consumer and advocates’ emphasis on social connectedness, however, very few studies explicitly focused on such approaches. Instead, the vast majority were based on manualised therapies designed for clinical settings, which typically focus on improving social skills instead of social connectedness itself.

These results were accordingly discussed during follow-up consultation with youth consumers and advocates. The importance of fostering social connectedness was re-emphasised by youth consumers and advocates, but the potential for more formal strategies to improve social skills was nonetheless still acknowledged. Thus, these and the additional perspectives of experts are interwoven throughout the review of more formal interventions below, while social connectedness interventions are highlighted as an area for future research.

### Prevention approaches

Mental health prevention approaches range from universal, community-based programmes designed for members of the general population, to selective approaches for those with known risk-factors, through to indicated (or early intervention) approaches for those showing early signs of disorder^25^. To date, the majority of preventative depression interventions targeting social relationships have been delivered in schools. There is also an emerging body of work focusing on online programmes.

#### School-based approaches

Schools are an ideal setting for programme implementation, facilitating cost-effective access to many young people in de-stigmatised and structured learning environments. Programmes are typically delivered in groups across several sessions, by teaching staff or external personnel. Although most programmes are based on cognitive behavioural therapy (CBT), many include components dedicated to social skills training^26^. Overall, these approaches attempt to prevent the onset or exacerbation of depressive symptoms by improving the resilience and coping skills of the individual, rather than working to improve social connections. In general, the efficacy of school-based programmes is supported by meta-analyses on both short- (0–6 months; *g* = 0.20) and long-term outcomes (>12 months; *g* = 0.1^27^). Effects were larger for targeted programmes (*g* = 0.32 vs. universal *g* = 0.19), and those delivered by external personnel (*g* = 0.30 vs. school staff *g* = 0.17)^27^. Below, we consider a range of empirically supported universal approaches that focus explicitly on improving social relationships.

LARS&LISA^1^ is a German programme based on the social information-processing model of social competence^28^. It has a dual cognitive and social focus, with social content aiming to foster adaptive social behaviour and increased social network use. This primarily occurs via assertiveness and social competence modules, where students apply learnt strategies via role-play within a group context (e.g., practicing non-avoidance, demonstrating verbal and non-verbal interest in others, sharing things about yourself). The programme has demonstrated effectiveness when delivered by psychologists rather than educators^29^, and groups are segregated by gender to promote authentic sharing. LARS&LISA prevented worsening of depressive symptoms in adolescents with minimal baseline symptoms, and reduced severity of symptoms in those with subthreshold depression over 6-month follow-up^30^. Programme effects are not moderated by comorbid anxiety and externalising symptoms^31^, but are moderated by self-efficacy and gender, favouring individuals with low self-efficacy at 3-month follow-up (*g* = 0.75)^32^, and women’s 12-month symptom trajectories^29,33^.

Strengthening Evidence base on scHool-based intErventions for pRomoting adolescent health (SEHER) is a multi-component intervention that seeks to promote a positive school climate (e.g., supportive relationships among community members, sense of school belonging, student social skills) at both the whole-of-school and individual level^34^. In a large randomised controlled trial of 74 secondary schools in India, students (aged 13–15 years; *N* = 15,232) who received this intervention reported significant improvements in school climate, attitudes toward gender equity, violence victimisation and perpetration, bullying and depressive symptoms. Promisingly, effects were largest at long-term follow-up (17 month), suggesting incremental benefits across time^34^. However, significant effects were only observed when the intervention was delivered by lay counsellors, not teachers.

MindOut is a teacher-led programme delivered in Ireland, designed to improve the social and emotional well-being of adolescents^35^. Content is structured around five core competencies: self-awareness, self-management, social awareness, relationship management and responsible decision-making. MindOut produced significant improvements in students’ (aged 15–18 years; *N* = 497) self-reported depressive symptoms and stress compared to controls^35^.

Strong Minds is a psychologist-led programme incorporating principles of acceptance and commitment therapy and positive psychology^36^. It includes dedicated social sessions, targeting assertiveness communication training and skills for developing and maintaining friends. In an Australian study, Strong Minds led to moderate improvements in subjective well-being across the sample (aged 15–18 years, *N* = 267, *d* = 0.43), and moderate reductions in self-reported depression (*d* = 0.53) for participants with subthreshold depressive symptoms at baseline^36^.

The Depression in Swedish Adolescents (DISA) programme includes cognitive behavioural components, communication training and social network development^37^. It is one of the few programmes to be evaluated when delivered by school personnel as part of regular curriculum, therefore providing ecological validity. Intervention participants reported significant improvements in self-reported depressive symptoms at 12-month follow-up compared to controls^37^.

Finally, The Connection Project aims to reframe peer relationships as sources of social support, rather than threat, to enhance school engagement and psychosocial functioning^38^. The programme is led by trained youth facilitators and has been shown to improve self-reported comfort with classmates among US secondary school students from marginalised communities (*N* = 610). At 4-month follow-up, students who received the intervention were rated as significantly more approachable than controls, had significantly increased their use of social supports, reported a significant reduction in depressive symptoms and increased their academic engagement. Of particular note, the use of social supports at follow-up was found to mediate depression and academic effects^38^.

Although these universal programmes have shown some efficacy in reducing depressive symptoms, it is generally unclear whether effects are driven by interpersonal improvements. For example, while the LARS&LISA programme theoretically works by promoting social competence and participation, mediation analyses found that neither the size of, nor frequency of engagement with, participants’ social networks mediated main effects for depressive symptoms^30^. Rather, the authors attributed their results to unmeasured effects, such as the influence of programme facilitators. There is also a need to trial the abovementioned programmes across diverse populations and cultural groups and, despite practical and economic advantages over externally delivered programmes, the efficacy of teacher-led programmes has yet to be firmly established^27^. Despite such caveats, the examples considered here provide preliminary evidence that socially-oriented, universal school-based programmes present a viable opportunity to prevent the worsening of depressive symptoms amongst young people in the general population.

In addition to universal approaches, one school-based, indicated programme focusing specifically on resolving interpersonal difficulties is interpersonal psychotherapy— adolescent skills training (IPT-AST). Based on IPT for depressed adolescents^39^, IPT-AST seeks to equip young people with the necessary interpersonal skills to buffer against known risk factors for depression (e.g., interpersonal conflict), while bolstering those that protect against onset (e.g., increased social support). Typically, IPT-AST comprises two individual pre-group sessions, where interpersonal goals are established, followed by eight group sessions. Group sessions involve psychoeducation about depression and interpersonal relationships, before participants are taught and encouraged to apply social and communication skills through role-plays and homework.

Horowitz and colleagues^40^ examined the efficacy of IPT-AST and a preventative cognitive-behavioural intervention in 380 high school students. IPT-AST produced a small reduction in depressive symptoms relative to no-intervention controls (*d* = 0.26). This effect was large (*d* = 0.84) when confined to adolescents with greater severity of subthreshold depressive symptoms at baseline. There were no significant differences between the active intervention groups, and effects did not persist at 6-month follow-up. In another school-based trial, 57 students with subthreshold depression were randomised to IPT-AST or standard school counselling^41^. Adolescents receiving IPT-AST reported significantly greater rates of change in depressive symptoms during treatment, fewer depressive symptoms, and better general functioning post-intervention relative to school counselling (*d* = 0.81–1.27). However, adolescents receiving school counselling continued to experience improvements through follow-up, rendering between-group comparisons non-significant at 12- and 18-month follow-up. In a subsequent trial using an active control condition matched for treatment frequency and duration^42^, the initial advantage of IPT-AST similarly dissipated, with no significant group differences evident at 24-month follow-up^43^.

Notably, high refusal rates were common in IPT-AST trials, indicating a need to address potential barriers (e.g., stigma) and limiting confidence in generalisability. Nonetheless, intervention satisfaction ratings were high, and attrition was low, suggesting good acceptability post-engagement^42^. IPT-AST treatment effects were not moderated by gender, age, ethnicity or income^42^. However, effects were moderated by severity of depressive symptoms, favouring those with higher baseline symptoms^40^.

IPT-AST does not seem to work as a universal prevention programme. Rather, positive results in universal samples are likely driven by improvements in high-risk adolescents^40^. Indeed, IPT-AST worked particularly well for those high in sociotropy, a personality trait characterised by strong investment in interpersonal relationships^40^. This was not the case for control or cognitive-behavioural groups, suggesting that IPT-AST might be more effective for young people who place high value on social relationships.

Although replication with larger trials and across diverse populations is required, the literature to date suggests that IPT-AST may be a promising early intervention strategy for young people experiencing high levels of subthreshold depressive symptoms. Combining IPT-AST with appropriate screening practices may therefore present a viable opportunity to prevent the onset of depressive disorders in educational settings. That being said, there is a need to examine the feasibility and effectiveness of IPT-AST when delivered by community-based staff (e.g., school counsellors or teachers).

#### Online approaches

Researchers have increasingly turned to the Internet to circumvent barriers associated with face-to-face intervention. By leveraging technological innovations and young people’s digital proclivities, online platforms afford anonymity and immediacy, while substantially increasing reach. Young people in our focus groups stressed the benefits of increased accessibility and inclusivity for marginalised or minority groups, commenting that online interventions afford a safe space to attract young people who might experience barriers to accessing traditional face-to-face interventions.

Online programmes are typically self-directed and module-based^44^. Most online programmes, such as MoodGym^45–47^, Thiswayup Schools^48^ and Master Your Mood Online^49^ include a variety of treatment modules or highly specific foci (e.g., how to cope with relationship breakups rather than improving relationships more generally), making it difficult to discern the specific effects of socially-focused modules on depressive symptomatology. Here, we turn to two programmes with a more explicit social focus: Smart, Positive, Active, Realistic, X-factor thoughts (SPARX-R)^50^ and Competent Adulthood Transition with Cognitive-behavioural, Humanistic and Interpersonal Training (CATCH-IT; 51).

SPARX-R is a preventative adaptation of SPARX, a gamified CBT intervention for adolescent depression^50^. SPARX-R users navigate a personalised avatar through a virtual environment where therapeutic content is delivered (e.g., behavioural activation and interpersonal skills)^52^. Preventative effects have been demonstrated in high achieving students (*N* = 540) leading up to final examinations; SPARX-R significantly reduced depressive symptoms post-intervention (*d* = 0.29) and at 6-month follow-up (*d* = 0.21) relative to attention-matched controls^52^. However, effects were not maintained at 18-month follow-up. Although there is a need to examine SPARX-R in a more general population, these positive effects observed in anticipation of a known stressor (i.e., school examinations) are promising.

CATCH-IT is an online intervention incorporating components of behavioural activation, CBT and IPT^51^. Intervention modules target vulnerability factors for depression (e.g., pessimistic appraisals and communication training), while strengthening those that protect against risk (e.g., activity scheduling, social skill and network development). As engagement in online interventions can be challenging, CATCH-IT has been examined in at-risk adolescents adjunct to primary care based engagement strategies: brief advice (BA; 1–2 min) or motivational interviewing (MI; 5–15 min)^51^. Both groups reported significant improvements in depression, interpersonal challenges and social support from peers, and the MI group were significantly less likely to experience a depressive episode through 12-month follow-up compared to the BA group^53–55^. Low self-efficacy and increased motivation for depression prevention at baseline predicted greater reduction in depressive symptoms at 2.5-year follow-up^56^. However, in a subsequent trial, CATCH-IT did not significantly reduce risk of a depressive episode in at-risk adolescents relative to Internet-based attention-controls^57^. Nevertheless, effects were significantly stronger for adolescents with greater severity of subthreshold depressive symptoms (up to 80% reduction in risk) compared to those with lower baseline scores. Depression outcome was not predicted by gender, age or ethnicity^57^. CATCH-IT has been culturally adapted for use in Arab Nations^58^, China^59^ and socio-economically disadvantaged African-American and Latino communities^60^.

Online mental health interventions are likely to play an increasingly central role in future preventative efforts. Results from existing socially-oriented programmes are promising, but multi-component programme designs preclude definitive conclusions about the effects of social components. Poor adherence remains a key challenge for online interventions, which may be improved by smart-phone based delivery.

### Treatment approaches

Beyond prevention approaches, a small number of interventions tailored towards improving young people’s social relationships focus on the treatment of full-threshold depressive disorders. Treatment approaches target the individual (improving interpersonal skills, addressing maladaptive thoughts and/or behaviours), but can also involve others in the individual’s treatment (e.g., family therapy, group interactions). To date, the literature suggests that interpersonal psychotherapy for adolescents (IPT-A) and family-focused interventions might be effective ways to treat depression in young people by seeking to improve their interpersonal relationships^61–65^.

#### Interpersonal psychotherapy for adolescents (IPT-A)

During interviews with clinical experts, IPT-A was frequently endorsed as the treatment of choice for youth depression. This time-limited, manualised psychotherapy seeks to reduce depressive symptoms by improving the quality of social relationships, recognising that interpersonal functioning and depression are closely linked, with problems in each domain perpetuating difficulties in the other^63^. Adapted from IPT for adults, IPT-A includes modifications such as a shortened treatment duration (16–20 to 12 weeks), inclusion of parents or caregivers and sensitivity to developmentally salient interpersonal issues, such as family conflict, the increasing need for individuation and bourgeoning autonomy, and development and maintenance of peer and romantic relationships^66^.

In the initial phase of IPT-A, the young person and therapist work collaboratively to explore and identify one of four problem areas to target during therapy: interpersonal disputes, role transitions, grief and interpersonal deficits. Through the middle phase, the therapist uses exploratory techniques, encouragement of affect, communication analysis and behaviour change techniques such as role-play and modelling, to help the young person develop interpersonal skills and affective understanding. In the termination phase, the therapist encourages reflection on progress and learnt skills and engages in relapse prevention planning.

The efficacy of IPT-A is supported by multiple meta-analyses^67–71^. These meta-analyses report on different outcomes, but it is important to acknowledge the overlap of included studies across them (i.e., of the five meta-analyses reported on below, 22 studies were included in total and 12 were included in more than one meta-analysis). Pu and colleagues (2017) analysed seven prospective randomised controlled trials (11–18 years; *N* = 538) and found that IPT-A was significantly more effective than controls in reducing depressive symptoms post-intervention and at follow-up (up to 12 months), and improving remission response post-intervention. IPT was also superior to controls in terms of improving quality of life and functioning. Sub-group and sensitivity analyses confirmed that IPT-A retained its advantage over controls irrespective of comparator type (waitlist, placebo or treatment as usual), depression definition (DSM-defined or symptom scale) and delivery format (individual, group or individual plus group).

Another meta-analysis of 20 IPT-A studies for 12–20 year olds (*N* = 910) showed large improvements in depressive symptoms post-intervention (*d* = 1.48) and through follow-up (20–52 weeks; *d* = 1.2)^67^. It also showed moderate and very large improvements in interpersonal functioning (*d* = 0.68), and general functioning (*d* = 2.85), respectively. Subgroup analyses showed that the main effect of treatment was attenuated when measured against active control conditions (*d* = 0.41). When compared to non-CBT active controls, IPT-A reduced depressive symptoms post-intervention with a medium effect (*d* = 0.64), while there was no significant advantage of IPT-A compared to CBT. However, this is inconsistent with three other meta-analyses: two network meta-analyses of several psychotherapies including IPT-A and CBT identified IPT-A as the best treatment for youth depression based on relative effect sizes of efficacy at post-intervention and long-term follow-up^71,72^; and a comprehensive meta-analysis of youth depression trials demonstrated greater treatment effects for IPT-A (*g* = 0.79) relative to CBT (*g* = 0.34)^68^.

Further evidence suggests that IPT-A is well suited and acceptable to young people. Meta-analyses show that IPT-A is associated with significantly fewer dropouts than IPT for adults (13.0 vs. 22.6%)^73^, and is either superior or non-inferior to active and non-active controls in terms of its all-cause discontinuation rate^67,70,71^. Although IPT-A trials have been predominantly conducted in the United States (*k* = 14), efficacy is also supported in low socioeconomic groups^74^, and in samples from Australia (*k* = 1), Canada (*k* = 1), Puerto Rico (*k* = 2), Uganda (*k* = 1) and Taiwan (*k* = 1)^67^. IPT-A has also been successfully implemented in community-based settings with internally trained school-based mental health professionals, where strongest treatment effects were observed for older adolescents (15–18 vs. 12–14 years) and those with greater severity of depressive symptoms at baseline^75^. Importantly, Zhou and colleagues found no evidence that participant characteristics significantly moderated treatment effects in their meta-analysis; however, effects were less pronounced in children and those with psychiatric comorbidities^71^. IPT-A thus appears to work well for most young people with depressive symptoms, but further work is needed to determine its utility in younger cohorts with more complex symptom profiles.

Despite strong evidence for its efficacy, reasons for such effects are only starting to become clear. Significant improvements in social functioning are often observed in IPT-A trials^75,76^. Although meta-analytic evidence has shown no significant differences between IPT-A and active controls in reducing interpersonal difficulties^67^, this may also be due to preferences in measurement; for example, Duffy and colleagues^67^ employed global measures of interpersonal functioning, rather than measures of specific relationships (e.g., peer conflict). Indeed, adolescents reporting higher levels of maternal conflict and social dysfunction with friends at baseline experienced significantly greater and more rapid reductions in depression following IPT-A relative to treatment as usual^77^. Moreover, IPT-A has been found to produce large reductions in attachment anxiety (*d* = 0.79) and avoidance (*d* = 0.83) in close relationships; reductions that were also significantly associated with improvements in depression^78^. Finally, improvements in peer and family interpersonal functioning partially mediated effects in depressed Latino youth, accounting for 38 and 62% of the indirect effect of IPT-A on depressive symptoms, respectively^79^. Sophisticated trial designs with theoretically grounded mediation analyses are needed to further examine putative change mechanisms of IPT-A. Nonetheless, the reviewed evidence suggests IPT-A might indeed facilitate symptomatic recovery via its action on interpersonal targets.

Altogether, IPT-A clearly presents a strong, evidence-based candidate for clinicians. Given the increased salience of interpersonal relationships throughout adolescence, its pragmatic, social orientation is likely to hold direct appeal for young people. Improvements in young people’s social relationships are also likely to afford some protection against future episodes: by improving quality of life and social functioning, IPT-A has the potential to break the cycle between low mood and interpersonal difficulties. Importantly, it also affords a sense of self-efficacy by actively engaging the young person with collaborative, social problem solving, reflection on progress and planning for relapse. In so doing, it might equip the young person with key skills to manage their mood and interpersonal difficulties in the future.

#### Family-focused treatment approaches

Despite the importance of interpersonal relationships in mitigating against depression in young people, relatively few studies have examined the efficacy of interventions within a family-focused context. In general, family-focused interventions assume that negative family environments (e.g., conflictual parent–child relationships, parental mental ill-health) prevent adolescents from developing the internal and interpersonal resources required to remain well when exposed to significant stressors. These approaches attempt to improve family relationships via psychoeducation and interpersonal communication building, which, in turn, can reduce depression and prevent recurrence.

A particularly noteworthy approach is attachment-based family therapy (ABFT), which is a brief (12–16 week) manualised therapy designed to repair or strengthen familial bonds and promote youth autonomy. The treatment includes five components: relational reframing, alliance building, parent education, reattachment and promoting competency^61,62^. Studies examining the efficacy of ABFT have yielded promising results; researchers have compared a 12-week ABFT programme to waitlist controls or treatment as usual in treating major depressive disorder (MDD) in adolescents^61,62^. ABFT was associated with large symptom reductions compared to control groups^61,62^. In one study, 81% of individuals receiving ABFT no longer met criteria for MDD at post-treatment, compared to substantially fewer (47%) in the control group^61^. Treatment effects were maintained 6 months later, with 87% of individuals continuing to no longer meet criteria for MDD. Further, ABFT was also associated with significant reductions in adolescent-perceived family conflict. While formal mediation analyses were not conducted, such results support the assumption that improving negative family environments, including conflict, might influence the course of MDD.

Studies examining the efficacy of family psychoeducation^64^ and systematic behaviour family therapy (SBFT; 65) have also demonstrated depressive symptom reductions in adolescents (13–18 years of age), but effects were non-significant. Nonetheless, Sanford et al. found that family psychoeducation was associated with significant improvements in social functioning and adolescent–parent relationships^64^. Dietz et al. also found that SBFT was associated with significant improvements in problem solving, and that such improvements were associated with a greater likelihood of remitting from MDD^65^. Thus, the non-significant effects of family psychoeducation and SBFT on MDD could have been due to temporal lags.

Overall, there is preliminary evidence in favour of ABFT as a treatment for depression in young people. Although ABFT—and potentially family psychoeducation and SBFT—likely work by improving depressogenic processes such as family conflict, social functioning, adolescent–parent relationships and problem solving, further research is required to confirm the efficacy of such interventions and their mechanisms of action.

#### Other treatment approaches

When interviewing clinical experts, a number of alternative, socially-oriented treatment approaches were highlighted, such as dialectical behaviour therapy^80^. However, there is a paucity of controlled trials examining their efficacy for depression in young people. In addition, more recent approaches that focus explicitly on improving social participation or connectedness, such as Groups 4 Health^81^ and social recovery therapy^82^, have yet to be trialled in populations of young people with depression.

According to our consultations with young people, online approaches hold particular promise, especially the use of social networking interventions like online support groups and moderated online social therapy. The latter approach combines peer support through online social networking, moderated by trained mental health professionals, with access to psychoeducation and individualised psychosocial interventions (e.g., the Rebound programme; 83). However, the efficacy of this approach remains to be established by rigorous controlled trials using samples of young people with depression, and the risk of exposing participants to potentially harmful interactions (e.g., cyberbullying) must also be borne in mind^44,84^. Nevertheless, online approaches are particularly well received by young people, increase their sense of connectedness with others, and reduce barriers to help-seeking^83^. There is also meta-analytic evidence indicating that digital mental health interventions can reduce depressive symptoms in clinical and non-clinical samples, particularly when programmes involve a high degree of therapist interaction^85^. Combining online delivery with traditional IPT-A might therefore present a viable means to deliver a highly accessible, youth-friendly, evidence-based intervention to young people with depression.

## Discussion

Our review revealed a number of approaches that may help to either prevent or treat depression in young people by facilitating improvements in the social domain. Certain school-based interventions have shown some promise as universal preventative approaches, while IPT-AST appears to be an effective indicated intervention for those experiencing greater severity of subthreshold depressive symptoms. There is also some evidence that socially-oriented online approaches might be effective for prevention, although this is still a nascent area.

When engaging with young people to discuss the outcomes of our review, school-based approaches received a lukewarm response, despite our positive findings. Their formalised nature was a notable disincentive, along with irrelevant content and competing time demands. Suggestions from young people to improve the efficacy of school-based programmes included delivery of successive programmes to allow for the consolidation of skills, and ensuring that content is inclusive for minority groups. Online approaches were more enthusiastically received. Their accessible and destigmatised nature were highlighted as attractive features, particularly for minority groups and those with social anxiety who might find it particularly challenging to attend or engage in face-to-face treatment.

With respect to treatment approaches, extensive evidence suggests that IPT-A is a highly effective psychotherapeutic strategy for young people experiencing depression. Echoing the recommendation of the World Health Organization^86^, the literature reviewed here supports the choice of IPT-A as a preferred treatment for young people with depression, particularly those experiencing interpersonal difficulties. ABFT has also attracted supportive findings, although further research is required before it can be reliably endorsed.

Of note, a major caveat applying to all of these approaches is variability in the quality of supportive studies. Greater use of randomised controlled trials, with larger samples, appropriate blinding and other measures to reduce risk of bias is warranted. Another outstanding question is whether socially oriented approaches are only well suited to those experiencing interpersonal difficulties. Although this makes intuitive sense, it is expected that improving one’s social relationships has a protective effect, regardless of the causes of depression. In any case, further research is required to shed light on this issue. Researchers have also tended to focus on the efficacy of interventions, rather than the mechanisms through which they operate. Dedicated designs (e.g., incorporating mediation and covariate analyses) are required to identify why different interventions work, and for whom they are likely to be effective.

In closing, involving young people in our research revealed a clear disconnect between the sorts of approaches they deemed important vs. the types of approaches reviewed here. Young people emphasised the need for informal ways to foster social connectedness and perceived social support, which were unequivocally preferred to formal strategies that improve social skills. Despite this feedback informing our literature search, we identified very few studies that explicitly focused on such approaches. Instead, the vast majority of studies—even school-based, group approaches—drew from manualised therapies designed for clinical settings, largely concentrating on improving social skills rather than social connectedness per se. A need to develop and explore the efficacy of approaches that aim to improve social connectedness, inclusion and participation cannot be understated.

## Supplementary information

Supplement 1

## Footnotes

1 Initially called ‘LISA’ (Leichtigkeit Im Sozialen Alltag), the programme was later revised to include the term ‘LARS’ (Lust An Realistischer Sicht) for identification with male participants, and an additional motivational module to encourage goal setting.
