# Supplementary material for: Facilitating improvements in young people’s social relationships to prevent or treat depression: A review of empirically supported interventions
Source: Transl Psychiatry. 2021 May 21;11:305. doi: 10.1038/s41398-021-01406-7 (PMC8139977; doi:10.1038/s41398-021-01406-7)
Supplement: Supplementary file 1 — Supplement 1 [file 41398_2021_1406_MOESM1_ESM.docx]

Supplement 1. Search terms employed within the Evidence Finder tool

| Line | Field | Search Term |
| --- | --- | --- |
| 1 | Mental health or substance use problem: | Depressive Disorders [DD] *OR* Bipolar Disorders [BP] |
| 2 | Stage of illness: | Universal Prevention [UP] *OR* At-Risk (indicated or selective prevention) [AR] *OR* Disorder Established (diagnosed disorder) [DE] *OR* Relapse Prevention [RP] *OR* Treatment Resistant/Treatment Refractory [TR] |
| 3 | Treatment / Intervention: | Psychological Intervention, any [PSY] *OR* Complementary & Alternative Intervention, any [CAM] *OR* Service Delivery & Improvement, any [SERV] |
| 4 | Design: | Randomised Controlled Trials [RCT] *OR* Controlled Clinical Trial [CCT] *OR* Systematic Review [SR] |
| 5 |  | 1 AND 2 AND 3 AND 4 |
